# Perceived social support and resilient mindset among Chinese undergraduates: the chain mediating roles of self-efficacy and meaning in life

**DOI:** 10.3389/fpsyg.2026.1882711

**Published:** 2026-09-18

**Authors:** Kuan-Ling Lin, Ying Chen

**Affiliations:** 1 School of Education, Fuzhou University of International Studies and Trade, Fuzhou, China; 2 School of Psychology, Zhejiang Normal University, Jinhua, China; 3 School of Art, Yango University, Fuzhou, China

**Keywords:** chain mediation model, meaning in life, perceived social support, resilient mindset, self-efficacy

## Abstract

**Objective:**

Grounded in Second Wave Positive Psychology (PP2.0), this study conceptualizes resilient mindset as a positive cognitive schema associated with the internalization of external relational and existential resources, rather than merely a static adaptive outcome. It examines how perceived social support is associated with resilient mindset among undergraduates through a sequential mechanism involving self-efficacy and meaning in life, thereby examining the proposed pathway from social resources to resilient cognition.

**Methods:**

A cross-sectional design was employed with 764 undergraduates recruited from four universities in China. Standardized instruments measuring perceived social support, self-efficacy, meaning in life, and resilient mindset were administered. Data were analyzed using SPSS 26.0 and Hayes’ PROCESS macro (Model 6). A bias-corrected bootstrap procedure with 5,000 resamples was used to test the sequential mediation model representing a “resource—agency—existential integration—schema formation” pathway.

**Results:**

Perceived social support significantly predicted resilient mindset. Both self-efficacy and meaning in life independently mediated this relationship. More importantly, a significant sequential mediation was identified, wherein self-efficacy was associated with meaning in life, which in turn was associated with resilient mindset. When mediators were included, the direct effect of perceived social support on resilient mindset became non-significant, indicating that the association is largely accounted for by the indirect pathways through self-efficacy and meaning in life.

**Conclusion:**

The findings advance PP2.0 by positioning resilience not as an outcome of adaptation, but as a higher-order cognitive associated with the integration of social and existential resources. The supported sequential pathway (“perceived social support → self-efficacy → meaning in life → resilient mindset”) specifies a theoretically integrated mechanism in which agency-based confidence is associated with existential meaning-making, jointly culminating in resilient cognition. This study shifts resilience research from outcome description to generative explanation, offering a structural model of resilient cognition under conditions of social uncertainty.

## 1 Introduction

The emergence of the new AI era, together with the structural uncertainty of post-pandemic society and the transformation of the global labor market, has placed undergraduates under new and complex psychological challenges. For instance, career anxiety has emerged as a significant barrier to life satisfaction in this era, a relationship that is profoundly influenced by an individual’s internal resources (Müceldili et al., 2023). Although these challenges are not unique to undergraduates, the university years represent an important developmental period as individuals transition from adolescence to adulthood and explore their identities, future directions, and increasing autonomy (Arnett, 2000). This transition also involves the movement from higher education toward employment, requiring young adults to develop career plans, new support systems, and adaptive resources (Halstead, 2021). Research with Chinese college students similarly highlights the importance of this period for academic, psychological, and career development (Yi et al., 2024). Traditional psychological perspectives have primarily conceptualized psychological resilience as an individual’s capacity to recover from adversity (Yıldırım and Tanrıverdi, 2020). However, such outcome-oriented definitions often overlook the cognitive transformation process that occurs during adversity itself. This limitation is particularly important when resilience is viewed not merely as an outcome of successful adaptation. Instead, it can be understood as a psychological orientation that shapes how individuals interpret and respond to adversity.

Within the development of second wave of positive psychology (PP2.0), psychological wellbeing is understood not simply as the absence of negative experiences or the pursuit of positive emotions, but as the capacity to confront, accept, and transform suffering in ways that support sustainable wellbeing (Wong, 2019; Wong et al., 2021). PP2.0 adopts a dialectical and existential perspective, viewing positive and negative experiences as interconnected and suffering as a potential pathway to meaning, resilience, and wellbeing. From this existential and dialectical perspective, positive and negative experiences are not necessarily treated as mutually exclusive. Instead, adversity may provide a context in which individuals mobilize meaning, inner resources, and self-transcendent capacities to cope with suffering and pursue psychological growth (Wong et al., 2021). Accordingly, resilience within PP2.0 extends beyond simply “bouncing back” from adversity and involves developing a resilient mindset that enables individuals to approach suffering as a condition that can be confronted, interpreted, and transformed (Arslan and Wong, 2024). Arslan and Wong (2024) further conceptualized resilient mindset as a cognitive orientation toward life’s challenges and developed the Resilient Mindset Scale to assess it. Recent evidence underscores that meaning in life and resilience act as vital buffers against psychological distress in this complex post-pandemic landscape (Baykal et al., 2026).

Against this background, the concept of a resilient mindset has been defined as a positive cognitive schema that determines how individuals reconstruct stressors (Arslan and Wong, 2024). They conceptualized resilient mindset within PP2.0 as a broader construct than traditional resilience, emphasizing a mindset that helps individuals embrace life’s challenges and develop adaptive responses to adversity (Arslan and Wong, 2024). Importantly, resilient mindset reflects a cognitive orientation that enables individuals to acknowledge adversity while responding with meaning and adaptive growth. This conceptualization is consistent with the central proposition of PP2.0 that sustainable psychological functioning does not require the elimination of suffering; rather, individuals may develop resilience by confronting difficult experiences, making meaning of them, and cultivating inner resources that support adaptive functioning (Wong et al., 2021). Recent work in positive existential psychology similarly argues that resilience can be understood as a striving to preserve and reconstruct the self through meaning-making when individuals encounter suffering and existential challenges (Churchill and Fisher-Smith, 2025). Nevertheless, the development of this mindset does not occur in isolation; resilience may reflect the interplay of multiple protective factors, including social support, cognitive resources, self-regulation, and positive self-perception (Abate et al., 2024). As an important external protective factors for undergraduates, perceived social support may be transformed into an internal cognitive defense mechanism. A recent meta-analysis confirmed a significant positive association between social support and meaning in life (Cai et al., 2026). Perceived social support has also been shown to reduce feelings of hopelessness by facilitating the construction of existential meaning (Zuo et al., 2021).

In this context, perceived social support may provide an important relational resource that can be internalized through motivational and existential processes. Self-efficacy may represent one motivational component of this internalization, whereas meaning in life may provide an existential framework through which individuals interpret adversity and personal experience. Thus, the present study considers the possibility that perceived social support is associated with resilient mindset through a progressive process involving resource perception, motivational activation, existential affirmation, and schema formation. This internal psychological pathway has become an important issue that warrants further investigation in contemporary psychology.

Although previous studies have extensively examined the relationship between social support and resilience, significant theoretical and empirical gaps remain. First, existing scholarship has long been dominated by outcome-oriented or functional perspectives on resilience, viewing it either as an adaptive outcome following adversity or as a relatively stable psychological trait (Yıldırım and Tanrıverdi, 2020). Such perspectives often neglect the cognitive processing mechanisms activated at the very moment adversity occurs. Therefore, the present study argues that it is necessary to clarify the essential nature of resilient mindset as a positive evaluative system from the perspective of cognitive schema theory, thereby compensating for the lack of cognitive-process analysis in traditional resilience research (Arslan and Wong, 2024). More specifically, resilient mindset shifts the focus from whether individuals recover from adversity to how they cognitively interpret and reconstruct it. This perspective is particularly compatible with PP2.0, which adds an existential and meaning-centered dimension to positive psychology by asking how individuals can transform unavoidable suffering into opportunities for meaning, growth, and self-transcendence (Wong, 2019; Wong et al., 2021).

Second, prior studies exploring protective factors have largely relied on conservation of resources theory. From the existential and dialectical perspective of PP2.0, fewer studies have examined how individuals construct deeper psychological defense systems through the acceptance and transformation of suffering (Arslan and Coşkun, 2024). From a positive existential perspective, resilience involves reconstructing one’s relationship with adversity through meaning and values (Churchill and Fisher-Smith, 2025). This aligns with the call for a “positive” existential psychology that views resilience as a striving to preserve the self through cognitive and value-based reconstruction (Churchill and Fisher-Smith, 2025). Thus, PP2.0 provides a theoretical basis for examining whether social resources are protective. It also helps explain how such resources may become psychologically meaningful through the acceptance, interpretation, and transformation of adversity. This theoretical gap has limited our understanding of how external social resources may be transformed into deeper internal psychological resources through existential and dialectical processes.

Finally, although the significance of self-efficacy and meaning in life for mental health has been widely confirmed (Zhao et al., 2023), few studies have integrated these two constructs into a single model to investigate how perceived social support may be associated with resilient mindset through the chain cascading effects of “motivation” (self-efficacy) and “value” (meaning in life). Empirical research suggests that self-efficacy and meaning in life often form a “winning combination” for student adjustment (Yuen and Datu, 2021), and self-efficacy has been identified as a critical precursor that fosters a sense of meaning (Zhao et al., 2023). Yuen and Datu (2021) also found associations between meaning in life and self-efficacy, highlighting their relevance to student adjustment. This sequence is theoretically consistent with the PP2.0 emphasis on the interaction between personal agency, meaning-making, and the transformation of adversity (Wong et al., 2021). From this perspective, motivational activation through self-efficacy may help individuals experience greater agency in confronting challenges, while existential affirmation through meaning in life may provide direction and value for interpreting those experiences. However, few studies have examined whether these processes operate sequentially in linking external social resources to resilient mindset. Accordingly, the present study proposes a chain-based process of “resource perception → motivational activation → existential affirmation → schema formation,” in which perceived social support represents an external relational resource, self-efficacy represents motivational agency, meaning in life represents existential meaning, and resilient mindset represents the resulting positive cognitive schema. This proposed sequence is a theoretically derived model informed by PP2.0 and previous empirical evidence, rather than a causal pathway established by PP2.0 itself. Addressing this gap represents the key point through which the present study seeks to extend and enrich the conceptualization of resilient mindset within the PP2.0 framework (Arslan and Wong, 2024).

## 2 Theoretical basis and hypothesis development

Grounded in PP2.0 and the conceptualization of resilient mindset proposed by Arslan and Wong (2024), this study examines how perceived social support is associated with resilient mindset through self-efficacy and meaning in life. Within PP2.0, resilience is viewed not simply as recovery from adversity, but as a process involving the confrontation and transformation of suffering through meaning-making and the mobilization of inner resources (Wong, 2019; Wong et al., 2021). Accordingly, resilient mindset is conceptualized as a positive cognitive schema through which individuals reinterpret and approach stress and challenges (Arslan and Coşkun, 2024). Thus, resilient mindset represents a more specific cognitive orientation toward adversity than an outcome-oriented conceptualization of psychological resilience.

Building on this perspective, the present study focuses on how external relational resources may be internalized through motivational and existential processes. Specifically, perceived social support may provide an important relational resource that is associated with individuals’ self-efficacy, while may in turn be associated with meaning in life. Together, these psychological processes may contribute to the development of resilient mindset. Accordingly, this study aims: (1) to clarify the conceptual distinction between resilient mindset and psychological resilience, thereby establishing the central role of resilient mindset as a positive cognitive schema; (2) to examine the independent mediating roles of self-efficacy and meaning in life in the relationship between perceived social support and resilient mindset; and (3) to construct and validate a chain mediation model of “perceived social support → self-efficacy → meaning in life → resilient mindset,” to examine how external relational resources may be associated with resilient cognition through motivational and existential processes.

### 2.1 The relationship between perceived social support and resilient mindset

From the perspective of PP2.0, individuals’ cognitive schemas are not formed in isolation, but are developed through dynamic interactions with environmental resources. As a core external resource within undergraduates’ psychological ecosystem, perceived social support may provide relational and psychological resources that support individuals’ responses to adversity (Arslan and Wong, 2024).

Perceived social support provides undergraduates with a fundamental sense of ontological security. Empirical studies have shown that perceived social support significantly reduces existential loneliness and feelings of insecurity among college students, thereby establishing a stable psychological safety boundary for coping with environmental uncertainty (Yang et al., 2026; Zuo et al., 2021). In the context of Chinese university students, perceived social support has been identified as a robust antecedent that significantly predicts resilience levels during academic stress (Hou et al., 2023). From a PP2.0 perspective, such supportive resources may create conditions in which individuals are more able to confront adversity rather than merely avoid or suppress it. This sense of security allows individuals to shift their cognitive appraisal from “threat-based avoidance” to “challenge-based acceptance,” which is consistent with the process through which resilient mindset may be cultivated (Arslan and Coşkun, 2024). When undergraduates perceive support from their social environment, they may be more likely to cognitively reconstruct adversity as a meaningful experience with potential for growth, thereby supporting the development of a resilient cognitive framework. Accordingly, this study proposes the following hypothesis:

*Hypothesis 1*: Perceived social support significantly and positively associated with resilient mindset.

### 2.2 The mediating effect of self-efficacy

Self-efficacy represents the core motivational component within the “cognition–motivation–existence” spiral. Within the proposed framework, self-efficacy may represent an important motivational mechanism through which perceived external resources are internalized into personal agency beliefs.

Specifically, the vicarious experiences and social persuasion provided by perceived social support may constitute important sources of self-efficacy. A growing body of empirical research consistently demonstrates that the higher the level of perceived social support perceived by undergraduates, the stronger their self-efficacy in overcoming academic and life-related challenges (Wang et al., 2024; Laksmiwati and Tondok, 2023). In this process, self-efficacy functions as a “motivational engine.” Individuals with high self-efficacy possess stronger beliefs in their ability to exert control when facing adversity, which may facilitate more adaptive attribution and appraisal processes at the cognitive level (Pillay et al., 2022; Martono et al., 2025). Through this motivational mechanism, external supportive resources may be progressively transformed into internalized volitional strength. In the present framework, self-efficacy is therefore viewed as an important, but not exclusive, mechanism through which perceived social support may move beyond the level of resource perception toward a deeper resilient cognitive schema. Accordingly, this study proposes the following hypothesis:

*Hypothesis 2*: Self-efficacy significantly mediates the relationship between perceived social support and resilient mindset.

### 2.3 The mediating effect of meaning in life

Meaning in life as an existential source of guidance, may function as an “existential engine” that provides direction and meaning for the development of resilient mindset. The PP2.0 framework emphasizes that psychological growth involves confronting suffering and discovering meaning rather than simply eliminating negative experiences (Wong, 2019; Wong et al., 2021). Within this perspective, existential affirmation provides a value-based basis through which individuals can interpret difficult experiences and integrate them into a broader understanding of life.

By fostering a sense of social connectedness among undergraduates, perceived social support enhances meaning in life by addressing fundamental questions of life purpose (Cai et al., 2026). Moreover, for Chinese college students, meaning in life is deeply intertwined with cultural identity and social connectedness, further reinforcing its role as a psychological energy core (Zhou et al., 2025). Although existing empirical scholarship has primarily focused on meaning in life as a predictor of traditional resilience outcomes (e.g., Li et al., 2025), this study argues that one important aspect of resilience within PP2.0 lies in the cognitive transformation of suffering. Undergraduates with a stronger sense of meaning are more capable of incorporating suffering into a growth-oriented framework (Arslan and Wong, 2024). Such existential affirmation may help resilient mindset become more than a temporary coping response by supporting a relatively stable cognitive logic oriented toward values, purpose, and adaptive growth. Accordingly, this study proposes the following hypothesis:

*Hypothesis 3*: Meaning in life significantly mediates the relationship between perceived social support and resilient mindset.

### 2.4 The chain mediating effect between self-efficacy and meaning in life

This study proposes a chain-based logical process: “resource perception → motivational activation → existential affirmation → schema formation”. Self-efficacy provides an important psychological basis for the development of meaning in life (Yuen and Datu, 2021). Recent empirical evidence among college students suggests that self-efficacy and meaning in life may operate sequentially in pathways linking positive experiences with psychological wellbeing (Guo et al., 2024). Enhanced self-efficacy enables individuals to perceive their actions as purposeful, which in turn may strengthen the development of motivational and existential resources (Zhao et al., 2023; Müceldili et al., 2023). Ultimately, this deep existential affirmation may contribute to a paradigm shift in cognitive appraisal, gradually supporting the consolidation of a stable resilient mindset (Arslan and Wong, 2024).

Accordingly, the present study constructs an interconnected psychological defense system in which perceived social support (resource) first may strengthen undergraduates’ efficacy beliefs (motivation), and this enhanced sense of agency subsequently may promote their understanding of life value and purpose (existence). Ultimately, this deep existential affirmation may contribute to a cognitive paradigm shift, gradually consolidating into a stable positive cognitive schema— resilient mindset. This pathway is consistent with the PP2.0 emphasis on confronting adversity, mobilizing inner resources, and transforming suffering through meaning-making and self-transcendence (Wong et al., 2021). At the same time, the specific sequence of perceived social support → self-efficacy → meaning in life → resilient mindset represents the integrative theoretical model proposed in the present study, rather than an established causal mechanism specified by PP2.0. Therefore, this study proposes the following hypothesis:

*Hypothesis 4*: Self-efficacy and meaning in life exert significant chain mediating effects in the relationship between perceived social support and resilient mindset.

Chain mediation describes a process in which two or more mediating variables operate in sequence, allowing the independent variable to exert an indirect effect on the dependent variable through a connected indirect pathway (Ye et al., 2016). Accordingly, a conceptual framework was developed based on the proposed hypotheses (Figure 1).

**Figure 1:**
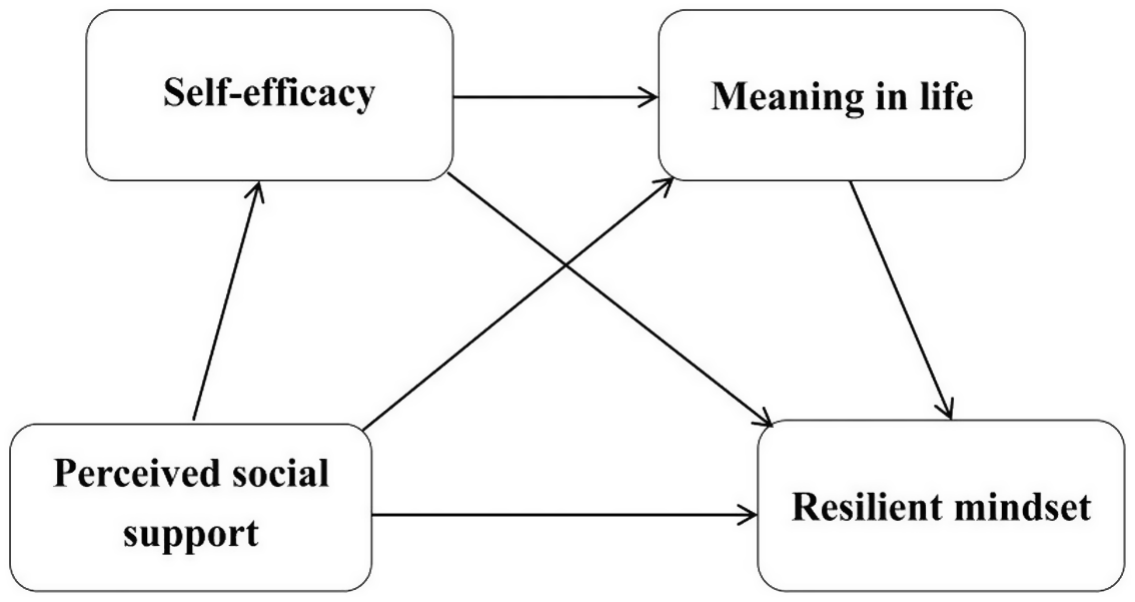
Hypothetical diagram of the conceptual framework.

## 3 Research methods

### 3.1 Participants

This study employed a convenience sampling method to recruit undergraduate students from four universities as research participants. A total of 878 questionnaires were collected. One duplicate submission was identified and removed, with the participant’s first response retained. Of the remaining 877 questionnaires, 73 failed an instructional attention check that required participants to select a specified response option and were excluded. An additional 40 questionnaires were excluded because of clear patterned responding, such as extended straight-lining or highly repetitive response patterns across questionnaire sections. No item-level missing responses were identified. The final analytic sample comprised 764 participants (87.0% of the initially collected questionnaires).

Participants ranged in age from 17 to 25 years (*M* = 19.92, SD = 1.29). Of the 764 participants, 255 were male (33.4%) and 509 were female (66.6%). In terms of academic disciplines, 266 participants (34.8%) majored in science and engineering, while 498 students (65.2%) majored in the humanities and social sciences. Regarding family structure, 130 participants (17.0%) were only children, whereas 634 (83.0%) had siblings. In terms of place of origin, 308 participants (40.3%) came from rural areas and 456 (59.7%) came from urban areas.

All participants voluntarily completed the survey based on informed consent. The questionnaires were administered anonymously to protect participants’ privacy. This study obtained ethical approval from the Ethics Committee of Zhejiang Normal University (ZSRT2025105).

### 3.2 Measures

#### 3.2.1 Perceived social support scale

The perceived social support scale was originally developed by Zimet et al. (1990), and the Chinese version used in this study was revised by Jiang and included in the standardized measurement compendium by Wang et al. (1999). It was used to assess participants’ subjective perception of social support. The scale consists of 12 items across three dimensions: family support, friend support, and significant other support. All items are rated on a 7-point Likert-type scale ranging from 1 (strongly disagree) to 7 (strongly agree), with higher total scores indicating higher levels of perceived social support. The Chinese version has been widely used in Chinese college student samples (Xiang et al., 2021). In this study, Cronbach’s alpha for the PSSS was 0.95.

#### 3.2.2 General self-efficacy scale

The General Self-Efficacy Scale was originally developed by Schwarzer and Jerusalem (1999) and later revised into a Chinese version by Wang et al. (2001). The scale consists of a single-dimensional structure with 10 items designed to assess individuals’ generalized confidence beliefs when facing environmental challenges. Responses were rated on a 4-point Likert scale ranging from 1 (“completely incorrect”) to 4 (“completely correct”). Total scores were calculated by summing all item responses, with higher scores indicating higher levels of self-efficacy. In the present study, the scale demonstrated good internal consistency reliability, with a Cronbach’s α coefficient of 0.94.

#### 3.2.3 Meaning in life questionnaire

The meaning in life questionnaire, originally developed by Steger et al. (2006) and revised into the Chinese version by Chen et al. (2015), is the most widely used standardized instrument for assessing individual sense of meaning in life, and was used to measure this construct in the current study. It comprises 10 items across two core dimensions: (1) Presence of Meaning, which assesses individuals’ perceived certainty and subjective experience of current meaning in life; (2) Search for Meaning, which measures individuals’ motivation and behavioral engagement in actively exploring and seeking meaning in life. All items are rated on a 7-point Likert-type scale ranging from 1 (strongly disagree) to 7 (strongly agree), with higher total scores indicating a stronger overall sense of meaning in life. In the current study, Cronbach’s alpha for the MLQ was 0.87.

#### 3.2.4 Resilient mindset scale

The resilient mindset scale, developed by Arslan and Wong (2024) based on the PP 2.0 framework, was used to measure participants’ resilient mindset. It assesses the core features of resilience-related cognition, including intentionality, flexibility, realism, and creativity. The scale comprises 6 items, all rated on a 5-point Likert-type scale ranging from 1 (strongly disagree) to 5 (strongly agree), with higher total scores reflecting higher levels of resilient mindset. The RMS has shown good reliability and validity across multiple empirical studies (Arslan, 2023; Arslan and Coşkun, 2024). In the current study, Cronbach’s alpha for the RMS was 0.95.

### 3.3 Statistical analysis

Data were analyzed using SPSS 26.0 and the PROCESS macro for SPSS. First, Harman’s single-factor test was conducted to assess the potential presence of common method bias. Descriptive statistics and Cronbach’s alpha coefficients were calculated, followed by Pearson correlation analyses among the study variables. Before the regression analyses, distributional characteristics were examined using skewness and kurtosis, homoscedasticity was assessed based on residual diagnostics, and multicollinearity was evaluated using tolerance and variance inflation factor (VIF) values. Because evidence of heteroscedasticity was observed in some regression models, heteroskedasticity-consistent standard errors (HC3) were used for regression inference. Finally, PROCESS Model 6 was applied to test the proposed chain mediation model, with gender, age, academic major, only-child status, and place of origin included as covariates. Indirect effects were estimated using 5,000 bias-corrected bootstrap samples with 95% confidence intervals.

## 4 Research results

### 4.1 Common method bias test

All data in this study were collected through self-report questionnaires administered to college students. Although procedural controls for common method bias were implemented during the survey process, such as expanding the geographical scope of sampling and using anonymous responses, it is still necessary to further examine its potential influence. To this end, Harman’s single-factor test was employed for statistical examination. An unrotated exploratory factor analysis was conducted on all items. The results showed that six factors with eigenvalues greater than 1 were extracted, with the first factor accounting for 37.20% of the variance, which is below the critical threshold of 40%. This indicates that there is no serious common method bias problem in this study.

### 4.2 Descriptive statistics and correlation analysis of perceived social support, self-efficacy, meaning in life and resilient mindset

Table 1 presents the descriptive statistics and Pearson correlation coefficients for perceived social support, self-efficacy, meaning in life, and resilient mindset among undergraduates. Skewness values ranged from −0.75 to 0.33, and kurtosis values ranged from 0.21 to 0.30, indicating no substantial deviations from normality. Pearson correlation analysis indicated that perceived social support was positively correlated with self-efficacy (*r* = 0.44, *p* < 0.01), meaning in life (*r* = 0.51, *p* < 0.01), and resilient mindset (*r* = 0.32, *p* < 0.01). Self-efficacy was also positively associated with meaning in life (*r* = 0.57, *p* < 0.01) and resilient mindset (*r* = 0.54, *p* < 0.01). In addition, meaning in life showed a significant positive correlation with resilient mindset (*r* = 0.54, *p* < 0.01). Overall, all study variables were significantly and positively correlated with one another, providing preliminary support for the proposed hypotheses.

**Table 1:** Descriptive statistics, distributional characteristics, and correlations.

Variables	*M*	SD	Skewness	Kurtosis	1	2	3
1. Perceived social support	5.06	1.01	−0.30	0.30			
2. Self-efficacy	2.56	0.59	0.33	0.21	0.44^**^		
3. Meaning in life	4.98	0.89	−0.01	0.29	0.51^**^	0.57^**^	
4. Resilient mindset	4.17	0.73	−0.75	0.24	0.32^**^	0.54^**^	0.54^**^

*N* = 764. **p < 0.01.

### 4.3 Regression analysis of perceived social support, self-efficacy and meaning in life on resilient mindset

Prior to the regression analyses, multicollinearity diagnostics showed that tolerance values ranged from 0.58 to 0.99 and variance inflation factor (VIF) values ranged from 1.02 to 1.72, indicating no problematic multicollinearity. Because evidence of heteroscedasticity was observed in some regression models, HC3-based heteroskedasticity-consistent standard errors were used for statistical inference. Table 2 presents the results of the separate regression analyses examining the associations of perceived social support, self-efficacy, and meaning in life with resilient mindset. Perceived social support was significantly and positively associated with resilient mindset (*B* = 0.23, *β* = 0.32, *t* = 8.69, *p* < 0.001), explaining 10% of the variance in resilient mindset (*R*^2^ = 0.10). Similarly, both self-efficacy (*B* = 0.67, *β* = 0.54, *t* = 18.32, *p* < 0.001, *R*^2^ = 0.29) and meaning in life (*B* = 0.44, *β* = 0.54, *t* = 19.46, *p* < 0.001, *R*^2^ = 0.29) were significantly and positively associated with resilient mindset. These results provide support for H1, indicating a significant positive association between perceived social support and resilient mindset among undergraduates.

**Table 2:** Separate regression analysis of perceived social support, self-efficacy, meaning in life on resilient mindset.

Variables	Resilient mindset					
	*B*	SE	*β*	*T*	*F*	*R* ^2^
Constant	3.01	0.14				
Perceived social support	0.23	0.03	0.32	8.69^***^	75.56^***^	0.10
Constant	2.46	0.10				
Self-efficacy	0.67	0.04	0.54	18.32^***^	335.63^***^	0.29
Constant	1.97	0.12				
Meaning in life	0.44	0.02	0.54	19.46^***^	378.75^***^	0.29

*N* = 764. *B*, unstandardized regression coefficient; *β,* standardized regression coefficient; SE, heteroskedasticity-consistent standard error based on the HC3 estimator; *R*^2^, proportion of variance explained. ***p < 0.001.

### 4.4 The chain mediating effects analysis

The SPSS PROCESS macro (Model 6) was employed to examine the chain mediating effects of self-efficacy and meaning in life in the relationship between perceived social support and resilient mindset. Gender, age, academic major, only-child status, and place of origin were included as control variables. Perceived social support was treated as the independent variable, self-efficacy and meaning in life as the mediating variables, and resilient mindset as the dependent variable. Table 3 presents the regression results of the mediation model. Using HC3, perceived social support was significantly and positively associated with self-efficacy (*B* = 0.25, *t* = 11.68, *p* < 0.001) and meaning in life (*B* = 0.29, *t* = 8.49, *p* < 0.001). Self-efficacy was also significantly and positively associated with meaning in life (*B* = 0.64, *t* = 13.04, *p* < 0.001). In the model with resilient mindset as the outcome, self-efficacy (*B* = 0.40, *t* = 8.97, *p* < 0.001), and meaning in life (*B* = 0.25, *t* = 9.22, *p* < 0.001) were significantly associated with resilient mindset, whereas the direct effect of perceived social support on resilient mindset was not significant (*B* = 0.03, *t* = 1.31, *p* = 0.189). Thus, although the overall association between perceived social support and resilient mindset was significant, its direct effect became non-significant after the mediators were included, indicating that the association was primarily accounted for by the indirect pathways in the specified model.

**Table 3:** Regression analysis of variable relationships in the mediation model (*N* = 764).

Variables		Overall fit coefficient			Regression coefficient significance		
Outcome variable	Predictor variable	*R*	*R* ^2^	*F*	*B*	SE	*t*
Self-efficacy	Perceived social support	0.47	0.22	28.82^***^	0.25	0.02	11.68^***^
Meaning in life	Perceived social support	0.65	0.42	67.51^***^	0.29	0.03	8.49^***^
	Self-efficacy				0.64	0.05	13.04^***^
Resilient mindset	Perceived social support	0.71	0.51	86.26^***^	0.03	0.02	1.31
	Self-efficacy				0.40	0.04	8.97^***^
	Meaning in life				0.25	0.03	9.22^***^

*B*, unstandardized regression coefficient; *R*^2^, proportion of variance explained. Heteroskedasticity-consistent standard errors based on the HC3 estimator are reported. ***p < 0.001.

Table 4 presents the total, direct, and indirect effects of perceived social support on resilient mindset. The total effect of perceived social support on resilient mindset was significant [effect = 0.24, SE = 0.02, 95% CI (0.20, 0.29)]. After self-efficacy and meaning in life were included, the direct effect was not significant [effect = 0.03, SE = 0.02, 95% CI (−0.02, 0.07)], accounting for 12.22% of the total effect. The total indirect effect was significant [effect = 0.21, SE = 0.02, 95% CI (0.18, 0.25)], accounting for 87.78% of the total effect.

**Table 4:** Total, direct, and indirect effects of perceived social support on resilient mindset (*N* = 764).

Effect pathway	Effect	SE	95% CI	Percentage
Total effect	0.24	0.02	[0.20, 0.29]	100%
Direct effect	0.03	0.02	[−0.01, 0.07]	12.22%
Total indirect effect	0.21	0.02	[0.18, 0.25]	87.78%
Perceived social support → self-efficacy → resilient mindset	0.10	0.01	[0.07, 0.13]	40.60%
Perceived social support → meaning in life → resilient mindset	0.07	0.01	[0.05, 0.10]	30.81%
Perceived social support →self-efficacy → meaning in life → resilient mindset	0.04	0.01	[0.03, 0.05]	16.37%

Effect, unstandardized effect estimate; SE, standard error; CI, confidence interval. HC3-based heteroskedasticity-consistent SEs are reported for total and direct effects; bootstrap SEs and bias-corrected 95% CIs are reported for indirect effects (5,000 bootstrap samples). Percentages indicate the proportion of the total effect.

Specifically, the indirect effect through self-efficacy (perceived social support → self-efficacy → resilient mindset) was significant [effect = 0.10, Boot SE = 0.01, 95% CI (0.08, 0.13)], accounting for 40.60% of the total effect and supporting H2. The indirect effect through meaning in life (perceived social support → meaning in life → resilient mindset) was also significant [effect = 0.07, Boot SE = 0.01, 95% CI (0.05, 0.10)], accounting for 30.81% of the total effect, supporting H3. In addition, the chain indirect effect through self-efficacy and meaning in life (perceived social support → self-efficacy → meaning in life → resilient mindset) was significant [effect = 0.04, Boot SE = 0.01, 95% CI (0.03, 0.05)], accounting for 16.37% of the total effect, supporting H4. These findings indicate that the association between perceived social support and resilient mindset was primarily accounted for by the separate and serial indirect pathways through self-efficacy and meaning in life. Figure 2 summarizes the serial mediation model and the corresponding unstandardized regression coefficients.

**Figure 2:**
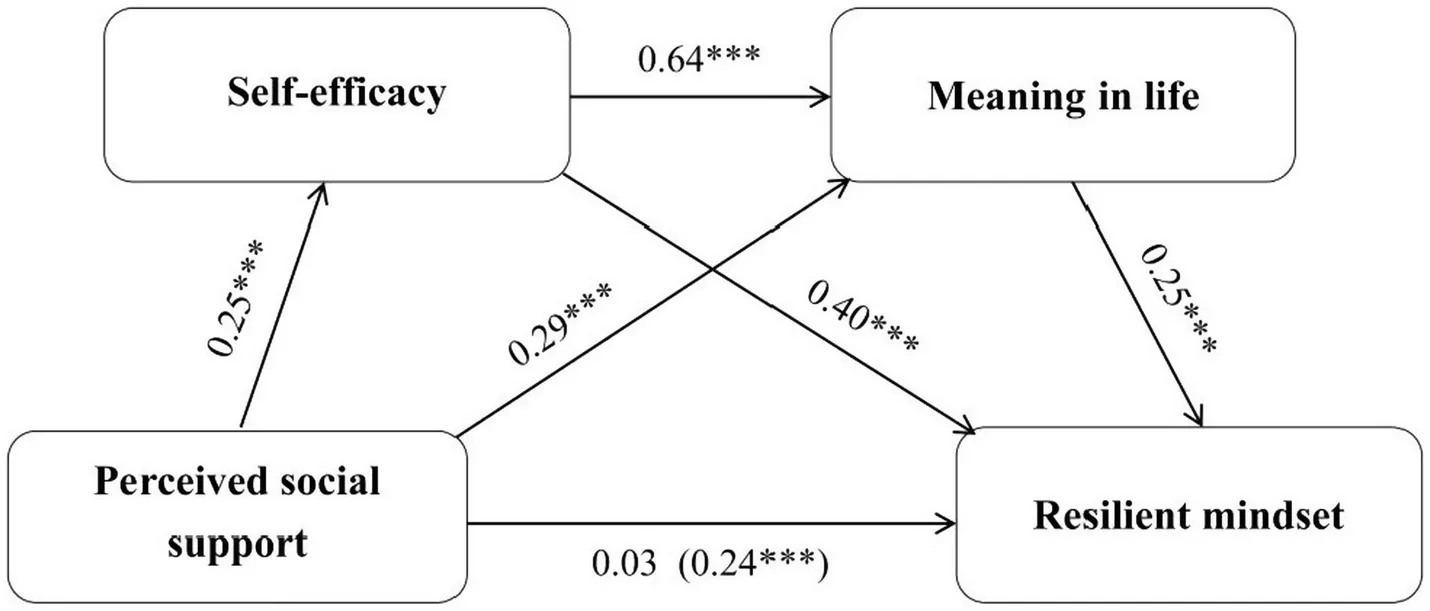
The chain mediation model of self-efficacy and meaning in life. The values along the paths represent unstandardized regression coefficients (*B*), and the value in parentheses represents the total effect of perceived social support on resilient mindset before the mediators were included. ***p < 0.001.

## 5 Discussion

### 5.1 The association between perceived social support and resilient mindset: from external nourishment to cognitive interpretation

This study found that perceived social support significantly and positively predicted resilient mindset among undergraduates, thereby supporting (H1). This finding indicates a significant positive association between perceived social support and resilient mindset, suggesting that students who perceive greater social support also tend to report stronger resilient mindsets. This finding not only reinforces the traditional view of perceived social support as a protective factor, but also deepens the understanding of its psychological function within the framework of PP2.0. Unlike traditional perspectives that conceptualize social support as a passive stress buffer, PP2.0 emphasizes the dialectical interaction between individuals and their environments (Arslan and Wong, 2024). For undergraduates navigating the transitional stage of emerging adulthood, perceived social support may provide an existential sense of psychological security. Such support is not merely a source of external resources; rather, it may also be associated with how individuals cognitively interpret their experiences. When individuals perceive stable social connectedness, they are more likely to reinterpret stressful experiences as meaningful existential challenges rather than purely destructive threats, thereby fostering the emergence of a resilient cognitive schema (Arslan and Wong, 2024; Ostafin and Proulx, 2020; Zuo et al., 2021). Accordingly, the findings suggest that the association between perceived social support and resilient mindset extends beyond emotional comfort and may be related to how individuals cognitively interpret and respond to adversity. Although the present study examined a theoretically specified directional pathway, the cross-sectional design does not rule out reciprocal or feedback processes between social support and resilient mindset.

### 5.2 The mediating roles of self-efficacy and meaning in life: motivational activation and existential affirmation

The findings further supported the independent mediating roles of self-efficacy (H2) and meaning in life (H3), revealing two critical psychological pathways associated with resilient mindset: the motivational pathway and the existential pathway. First, self-efficacy functions as a “motivational engine” that may help explain how external resources are associated with personal agency beliefs. Perceived social support may be associated with stronger individuals’ perceived capability to cope with difficulties by providing emotional encouragement and supportive interpersonal experiences (Laksmiwati and Tondok, 2023). These efficacy beliefs may be associated with a greater tendency among undergraduates to actively adopt cognitive reconstruction strategies when facing adversity rather than falling into learned helplessness. In this sense, self-efficacy may represent a psychological mechanism through which perceived environmental support is linked to action-oriented confidence and resilient cognition.

Second, meaning in life functions as an “existential engine” that may provide a sense of direction and purpose that is relevant to resilient mindset. According to the core logic of PP2.0, psychological wellbeing is not merely reflected in symptom reduction, but in individuals’ capacity to construct meaning through suffering (Wong, 2020; Wong et al., 2021). Longitudinal evidence provided by Li et al. (2025) further suggests that meaning experience is associated with subsequent resilience-related outcomes. When undergraduates establish a clear sense of life purpose and value, their cognitive systems acquire a deeper motivational orientation toward growth and hope. Consequently, resilient mindset may be understood as being associated with a more enduring cognitive orientation grounded in existential meaning.

### 5.3 The theoretical significance of the chain mediation: a proposed pathway from social resources to resilient cognition

The most important theoretical contribution of the present study lies in supporting the sequential pathway through which external resources are linked to internal cognitive schemas. This finding is consistent with the upward “cognition–motivation–existence” spiral mechanism proposed within the PP2.0 framework (Arslan and Wong, 2024; Wong et al., 2021). Within this mechanism, resilient mindset is no longer conceptualized as an innate personality trait or a simple recovery outcome following adversity. Instead, the findings are consistent with conceptualizing resilient mindset as a positive cognitive schema that may be associated with social and psychological resources such as perceived social support, self-efficacy, and meaning in life (Arslan and Coşkun, 2024; Yıldırım and Tanrıverdi, 2020).

More specifically, this proposed pathway illustrates how psychological defense mechanisms may extend beyond instrumental coping processes into deeper existential systems. Self-efficacy, as the “motivational engine,” addresses the issue of personal agency in the face of adversity, whereas meaning in life, as the “existential engine,” answers fundamental questions concerning life value and purpose. The sequential integration of these two mechanisms may enable undergraduates to elevate temporary feelings of competence into enduring experiences of existential value (Müceldili et al., 2023; Yuen and Datu, 2021). Such existential affirmation may be associated with a paradigm shift in cognition, consolidating positive evaluative logic into a more adaptive cognitive appraisal system—that is, resilient mindset itself (Arslan and Wong, 2024; Li et al., 2025).

The significant of this chain mediating effect not only statistically explains the interactions among the study variables, but also theoretically reveals the potential logic through which resilient mindset functions as a “psychological firewall.” Specifically, external supportive resources may be associated with the development of a stable underlying cognitive framework. This process provides a rigorous theoretical explanation for how undergraduates may move from mere survival toward psychological flourishing within environments characterized by structural uncertainty (Arslan and Wong, 2024; Zhao et al., 2023).

## 6 Implications, limitations and future research

By confirming the sequential mediation pathway of “perceived social support → self-efficacy → meaning in life → resilient mindset,” the present study provides empirical support for the proposed sequential association within the framework of PP2.0. The primary theoretical contribution lies in advancing resilience research from an “outcome-oriented” perspective toward a “generative-oriented” perspective (Arslan and Wong, 2024). The findings suggest that resilient mindset is not merely an accumulation of isolated psychological resources, but rather may be understood as a positive cognitive schema associated with the integration of multiple psychological resources. Specifically, external nourishment (perceived social support) may be linked to the internalization of resources through the motivational system (self-efficacy), and this motivational process may be further associated with existential affirmation through the existential system (meaning in life), which, in turn, may be associated with resilient mindset as a positive cognitive schema (Li et al., 2025). This pattern is consistent with the possibility that, among undergraduates facing structural uncertainty, the integration of motivational and existential resources may be associated with more adaptive cognitive orientations toward adversity.

The present study also provides practical guidance for mental health education in higher education settings. First, educators should recognize not only the resource function of perceived social support, but also the importance of the “internalization efficiency” of such resources. External support alone, without the activation of individual agency through self-efficacy, may be insufficient to develop a more adaptive psychological response to adversity. To enhance this internalization process, educators could incorporate brief peer-support reflection activities, such as asking students to identify available sources of support, discuss how they have used these resources to cope with a recent difficulty, and develop specific action plans for seeking support when facing future challenges. Second, intervention strategies should move beyond the simple reduction of negative emotions and place greater emphasis on deeper processes of meaning reconstruction. Universities should encourage undergraduates to accept the uncontrollable aspects of life and, through Meaning Therapy, develop a deeper understanding of life value and existential purpose. For example, meaning-oriented group sessions could use guided reflection on stressful experiences, personal values, and future goals to help students connect external support with their own coping efforts and life purpose. Such existential affirmation may be associated with more adaptive cognitive orientations toward adversity. This schema-oriented intervention approach may help undergraduates move from fragile recovery toward more adaptive and sustainable coping.

Despite providing important empirical evidence regarding the proposed associations underlying resilient mindset among undergraduates, the present study still contains several methodological limitations that warrant further refinement. First, the study adopted a cross-sectional design. Although a theoretically rigorous chain mediation model was constructed based on the PP2.0 framework and the proposed pathways were statistically supported, this static form of data collection remains insufficient for fully establishing the temporal causal relationships among variables. Future studies should therefore employ experimental or longitudinal approaches to further strengthen the generative logic proposed in this study (Li et al., 2025; Zhou et al., 2025). Second, all data were collected through self-report measures. Although the results of the common method bias test indicated that bias remained within an acceptable range, the highly subjective nature of constructs such as meaning in life and resilient mindset makes it difficult to completely eliminate the potential influence of social desirability and cognitive bias on the validity of the findings. Finally, the sample was limited to undergraduate students. Although this population is highly representative due to its transitional developmental stage characterized by psychological separation and identity reconstruction, caution should still be exercised when generalizing the findings to individuals at other life stages, such as early-career adults, middle-aged adults, or older populations.

To address these limitations, future research should advance theoretical and empirical development through three major directions: dynamic longitudinal investigation, experimental intervention, and cross-cultural comparison. First, future studies are encouraged to adopt longitudinal designs, such as cross-lagged panel analyses, to dynamically examine the temporal ordering and longitudinal associations of the proposed pathway of “resource internalization → motivational activation → existential affirmation → schema consolidation” within real-life stressful experiences. Such approaches would provide a stronger empirical foundation for the generative perspective of resilient mindset. Second, future research may integrate Meaning Therapy or cognitive-behavioral interventions into randomized controlled trials to investigate whether enhancing individuals’ experiences of meaning in life can contribute to more adaptive cognitive orientations toward adversity, thereby further validating the practical and educational applicability of the present model within university mental health education. Finally, given that the PP2.0 framework emphasizes the dialectical and culturally embedded understanding of suffering, cross-cultural comparative studies may help determine whether the mechanisms through which social support is transformed into resilient mindset differ across cultural contexts. Such investigations may contribute to the development of a more culturally sensitive and universally applicable model of resilient cognitive development among undergraduates (Arslan and Wong, 2024; Yang et al., 2026).

## 7 Conclusion

Grounded in the framework of PP2.0, the present study provides evidence that perceived social support is associated with undergraduates’ resilient mindset through the independent and sequential mediating roles of self-efficacy and meaning in life. More importantly, the findings advance resilience research from an outcome-oriented perspective toward a generative perspective by conceptualizing resilient mindset as a positive cognitive schema rather than merely a recovery outcome following adversity. The supported “resource–motivation–existence–schema” pathway further illustrates how external supportive resources are linked to motivational activation and existential affirmation, ultimately associated with resilient mindset as a positive cognitive schema. In this sense, resilient mindset reflects not simply an adaptive response to stress, but an adaptive cognitive orientation through which adversity may be viewed as an opportunity for growth. These findings provide empirical support for the PP2.0 proposition that psychological flourishing emerges through the dialectical integration of suffering, meaning, and positive reconstruction, thereby extending current theoretical understanding of how resilient cognition develops under conditions of structural uncertainty.

## Data Availability

The raw data supporting the conclusions of this article will be made available by the authors, without undue reservation.

## References

[ref1] Abate B. B. Sendekie A. K. Tadesse A. W. Engdaw T. Mengesha A. Zemariam A. B. . (2024). Resilience after adversity: an umbrella review of adversity protective factors and resilience-promoting interventions. Front. Psych. 15:1391312. doi: 10.3389/fpsyt.2024.1391312, 39429523 PMC11487322

[ref2] Arnett J. J. (2000). Emerging adulthood: a theory of development from the late teens through the twenties. Am. Psychol. 55, 469–480. doi: 10.1037/0003-066X.55.5.469, 10842426

[ref3] Arslan G. (2023). My parents inspire me to succeed: enhancing academic well-being through resilient mindset and mental toughness. Child Indic. Res. 16, 2475–2488. doi: 10.1007/s12187-023-10063-9

[ref4] Arslan G. Coşkun M. (2024). Coronavirus-related stressors, resilient mindset, loneliness, depressive symptoms in college students: testing a moderated mediation model. Psychol. Rep. 127, 1633–1651. doi: 10.1177/00332941221139721 36377653

[ref5] Arslan G. Wong P. T. P. (2024). Embracing life’s challenges: developing a tool for assessing resilient mindset in second wave positive psychology. J. Happiness Health 4, 1–10. doi: 10.47602/johah.v4i1.53

[ref6] Baykal E. Contreras F. Abid G. Aldeanueva-Fernández I. (2026). Well-being in the post-pandemic era: the role of meaning in life and resilience. Front. Psychol. 16:1696860. doi: 10.3389/fpsyg.2025.1696860, 41657824 PMC12873481

[ref7] Cai J. Y. Wang X. Q. Zhang T. Pan S. Y. (2026). Social support and meaning in life: a meta-analysis. J. Happiness Stud. 27, 23–47. doi: 10.1007/s10902-026-01008-4

[ref8] Chen W. Ge Y. Hu Y. Zhang J. (2015). Applicability and generalizability of the revised meaning in life questionnaire: based on classical test theory and multidimensional Rasch model. Chin. J. Clin. Psychol. 23, 604–607, 614. doi: 10.16128/j.cnki.1005-3611.2015.04.009

[ref9] Churchill S. D. Fisher-Smith A. (2025). Psychological resilience as striving to preserve oneself: on the possibility of a “positive” existential psychology. J. Posit. Psychol. 20, 811–822. doi: 10.1080/17439760.2025.2509984

[ref10] Guo Y. Qin K. Yu Y. Wang L. Xu F. Zheng Q. . (2024). Physical exercise can enhance meaning in life of college students: the chain mediating role of self-efficacy and life satisfaction. Front. Psychol. 14:1306257. doi: 10.3389/fpsyg.2023.1306257, 38274680 PMC10808599

[ref11] Halstead T. J. (2021). “Requisite metamorphoses: college graduates transitioning from school to work,” in Young adult Development at the school-to-work transition: International Pathways and Processes, eds. Marshall E. A. Symonds J. E. (New York: Oxford University Press), 443–460.

[ref12] Hou Y. Zhang Y. Cao X. Lei G. Liu G. (2023). The association between perceived social support and resilience among Chinese university students: a moderated mediation model. Psychol. Sch. 61, 1474–1490. doi: 10.1002/pits.23122

[ref13] Laksmiwati E. D. Tondok M. S. (2023). Perceived social support, academic self-efficacy, and anxiety among final year undergraduate students: a mediation analysis. Bull. Counseling Psychother. 5, 173–182. doi: 10.51214/00202305514000

[ref14] Li D. Geng X. Yan X. Gao S. (2025). Relationship between meaning in life and resilience: a longitudinal study and a randomised controlled trial intervention. Int. J. Psychol. 60:e70077. doi: 10.1002/ijop.70077 40641127

[ref15] Martono S. Prajanti S. D. W. Purwanta E. Dwiningrum S. I. A. (2025). The role of mental toughness and positive psychological capital in the effect of perceived social support on student resilience. Educ. Process Int. J. 16:e2025277. doi: 10.22521/edupij.2025.16.277

[ref16] Müceldili B. Tatar B. Erdil O. (2023). Career anxiety as a barrier to life satisfaction among undergraduate students: the role of meaning in life and self-efficacy. Int. J. Educ. Vocat. Guid. 25, 607–631. doi: 10.1007/s10775-023-09617-8

[ref17] Ostafin B. D. Proulx T. (2020). Meaning in life and resilience to stressors. Anxiety Stress Coping 33, 603–622. doi: 10.1080/10615806.2020.1800655, 32755239

[ref18] Pillay D. Nel P. Van Zyl E. (2022). Positive affect and resilience: exploring the role of self-efficacy and self-regulation. A serial mediation model. S. Afr. J. Ind. Psychol. 48:a1913. doi: 10.4102/sajip.v48i0.1913

[ref19] Schwarzer R. Jerusalem M. (1999). Skalen zur Erfassung von Lehrer- und Schülermerkmalen: Dokumentation der psychometrischen Verfahren im Rahmen der Wissenschaftlichen Begleitung des Modellversuchs Selbstwirksame Schulen. Berlin, Germany: Freie Universität Berlin.

[ref20] Steger M. F. Frazier P. Oishi S. Kaler M. (2006). The meaning in life questionnaire: assessing the presence of and search for meaning in life. J. Couns. Psychol. 53, 80–93. doi: 10.1037/0022-0167.53.1.80

[ref21] Wang C. Hu Z. Liu Y. (2001). Evidences for reliability and validity of the Chinese version of general self-efficacy scale. Chin. J. Appl. Psychol. 7, 37–40. doi: 10.3785/1006-6020.2001.01.0037

[ref22] Wang X. Wang X. Ma H. (1999). Handbook of Mental Health Assessment Scales. Revised Edn Beijing: Chinese Journal of Mental Health, 131–133.

[ref23] Wang X. Wang Y. Ye Y. (2024). Subjective socioeconomic status predicts e-learning engagement in college students: the mediating role of perceived social support and self-efficacy. Eur. J. Psychol. Educ. 39, 1119–1134. doi: 10.1007/s10212-023-00725-1

[ref24] Wong P. T. P. (2019). Second wave positive psychology’s (PP 2.0) contribution to counselling psychology. Couns. Psychol. Q. 32, 275–284. doi: 10.1080/09515070.2019.1671320, 37339054

[ref25] Wong P. T. P. (2020). Existential positive psychology and integrative meaning therapy. Int. Rev. Psychiatry 32, 565–578. doi: 10.1080/09540261.2020.1814703, 33016788

[ref26] Wong P. T. P. Mayer C.-H. Arslan G. (2021). Editorial: COVID-19 existential positive psychology (PP2.0): the new science of self-transcendence. Front. Psychol. 12:800308. doi: 10.3389/fpsyg.2021.800308 PMC8699172 34956025

[ref27] Xiang G. Teng Z. Guo C. Chen H. Miao H. (2021). Parent-adolescent attachment and peer attachment associated with hope: a longitudinal study of first-year college students. Psychol. Dev. Educ. 37, 371–380. doi: 10.16187/j.cnki.issn1001-4918.2021.03.08

[ref28] Yang Z. Yang Z. Wen W. Zeng Q. (2026). Perceived social support and meaning in life of adolescents: roles of psychological insecurity and psychological capital. Curr. Psychol. 45:363. doi: 10.1007/s12144-025-08967-6

[ref29] Ye L. Wang B. Ge Y. Bu S. Wang L. (2016). The effect of athletes' gratitude on burnout: chain mediating effects of social support and mental toughness. Chin. Sport Sci. 36, 39–49. doi: 10.16469/j.css.201611005

[ref30] Yi C. Wei J. Ti Y. Li J.-B. (2024). Trajectories of self-control in emerging adulthood: implications for academic, psychological, and career development among Chinese college students. Appl. Res. Qual. Life 19, 641–664. doi: 10.1007/s11482-023-10258-w

[ref31] Yıldırım M. Tanrıverdi F. Ç. (2020). Social support, resilience and subjective well-being in college students. J. Posit. Sch. Psychol. 5, 127–135. doi: 10.47602/jpsp.v5i2.229

[ref32] Yuen M. Datu J. A. D. (2021). Meaning in life, connectedness, academic self-efficacy, and personal self-efficacy: a winning combination. Sch. Psychol. Int. 42, 1–21. doi: 10.1177/0143034320973370

[ref33] Zhao H. Zhang M. Li Y. Wang Z. (2023). The effect of growth mindset on adolescents' meaning in life: the roles of self-efficacy and gratitude. Psychol. Res. Behav. Manag. 16, 4647–4664. doi: 10.2147/PRBM.S428397, 38024657 PMC10655738

[ref34] Zhou S. Jiang L. Li W. Leng M. (2025). Meaning in life from a cultural perspective: the role of cultural identity, perceived social support, and resilience among Chinese college students. Humanit. Soc. Sci. Commun. 12:91. doi: 10.1057/s41599-025-04424-8

[ref35] Zimet G. D. Powell S. S. Farley G. K. Werkman S. Berkoff K. A. (1990). Psychometric characteristics of the multidimensional scale of perceived social support. J. Pers. Assess. 55, 610–617. doi: 10.1080/00223891.1990.9674095, 2280326

[ref36] Zuo B. Yang K. Yao Y. Han S. Nie S. Wen F. (2021). The relationship of perceived social support to feelings of hopelessness under COVID-19 pandemic: the effects of epidemic risk and meaning in life. Pers. Individ. Dif. 183:111110. doi: 10.1016/j.paid.2021.111110, 34511679 PMC8416552

